# From the Editor's Desk

**Published:** 2017

## Introduction

Call me old fashioned if you wish, but I remain saddened by the apparent erosion of clinical skills in cardiology and the determined rush to involve ever more-sophisticated imaging modalities prior to simple clinical evaluation, examination of a chest radiograph and an ECG. The ECG remains one of the oldest ways of evaluating the heart; it is cheap and non-invasive and when interpreted correctly, can contribute an enormous amount of information. In this issue of the journal, Viljoen and colleagues (page 257) provide an example of the utility of the simple resting 12-lead ECG in explaining the patient’s symptoms and prompting appropriate treatment. If this ECG abnormality had been recognised at primary or secondary level, the patient could have been referred promptly. Instead, he joined the queue for unnecessary echocardiography. This will, I hope, be the firstin an ECG series, of which each will consist of a brief clinical vignette, an ECG and an explanation linking the clinical features to the ECG.

It is a particular pleasure to write this piece to introduce the articles in this issue. All of them emanate from Africa and all address common and important diseases of Africa. On page 262, Dzudie and his colleagues from PASCAR outline a roadmap for hypertension in Africa. The intention is to develop practical guidelines on how to implement strategies that translate existing knowledge into effective action and improve detection, treatment and control of hypertension in sub-Saharan Africa by the year 2025. Given the importance of hypertension to cardiovascular disease in Africa, successful adoption of such a roadmap should make a major contribution to health on the continent.

Rheumatic heart disease remains a problem in Africa. Given the scarcity of surgical facilities, prevention and secondary prophylaxis remain at the heart of attempts to control the condition, and Long and colleagues (page 242) describe an initiative in Zambia, consisting of an educational and accessto- medicine programme aimed at increasing appropriate use of benzathine penicillin for the prevention and management of rheumatic heart disease. They describe well-established barriers to correct care, including the concerns of healthcare workers. Gratifyingly, they are able to show a measure of success, as indicated by increased use of benzathine penicillin.

A programme such as that described by Long will only succeed on a large scale if it is incorporated into an effective primary care network such as that envisaged for Africa by Ojji and co-authors (page 251). Such a programme requires effective political will and has substantial financial implications but remains essential.

Continuing the theme of rheumatic heart disease, in a retrospective record review, Makrexeni and colleagues describe the spectrum of rheumatic heart disease at a paediatric cardiology tertiary care service in Port Elizabeth, South Africa (page 248). Sadly, the majority presented with established structural heart disease and a minority with acute rheumatic fever, which would allow institution of effective prophylaxis. Such retrospective series are often criticised but they do serve the important role of reminding us of the reality of our day-to-day practice.

Meel and co-authors (page 215), using echocardiographic evaluation of a contemporary cohort of patients with rheumatic mitral regurgitation, describe similar changes to the presentation at another institution, with few patients having acute rheumatic fever, most being older and characterised by more co-morbidities and thickened and calcified valves.

Endomyocardial fibrosis remains a rare and poorly understood condition with striking geographic variability in its distribution. Khalil and co-authors present an analysis of their echocardiographic experience (page 208) and provide echocardiographic images, which should be helpful to all performing imaging in this condition.

It is a great privilege to be able to publish in this journal the work of African authors reporting their experiences with important diseases of Africa.

